# A robust computational quest: Discovering potential hits to improve the treatment of pyrazinamide‐resistant *Mycobacterium tuberculosis*

**DOI:** 10.1111/jcmm.18279

**Published:** 2024-04-17

**Authors:** Muhammad Shahab, Gabriel Christian de Farias Morais, Shopnil Akash, Umberto Laino Fulco, Jonas Ivan Nobre Oliveira, Guojun Zheng, Shahina Akter

**Affiliations:** ^1^ State key laboratories of Chemical Resources Engineering Beijing, University of Chemical Technology Beijing China; ^2^ Department of Biophysics and Pharmacology, Bioscience Center Federal University of Rio Grande do Norte Natal Rio Grande do Norte Brazil; ^3^ Department of Pharmacy Daffodil International University Dhaka Bangladesh; ^4^ Bangladesh Council of Scientific and Industrial Research Dhaka Bangladesh

**Keywords:** ADMET, docking, molecular dynamic, molecular modelling, *Mycobacterium tuberculosis*, pharmacoinformatic, pharmacophore‐based virtual screening, pyrazinamide resistance, quantum chemical calculations

## Abstract

The rise of pyrazinamide (PZA)‐resistant strains of *Mycobacterium tuberculosis* (MTB) poses a major challenge to conventional tuberculosis (TB) treatments. PZA, a cornerstone of TB therapy, must be activated by the mycobacterial enzyme pyrazinamidase (PZase) to convert its active form, pyrazinoic acid, which targets the ribosomal protein S1. Resistance, often associated with mutations in the RpsA protein, complicates treatment and highlights a critical gap in the understanding of structural dynamics and mechanisms of resistance, particularly in the context of the G97D mutation. This study utilizes a novel integration of computational techniques, including multiscale biomolecular and molecular dynamics simulations, physicochemical and medicinal chemistry predictions, quantum computations and virtual screening from the ZINC and Chembridge databases, to elucidate the resistance mechanism and identify lead compounds that have the potential to improve treatment outcomes for PZA‐resistant MTB, namely ZINC15913786, ZINC20735155, Chem10269711, Chem10279789 and Chem10295790. These computational methods offer a cost‐effective, rapid alternative to traditional drug trials by bypassing the need for organic subjects while providing highly accurate insight into the binding sites and efficacy of new drug candidates. The need for rapid and appropriate drug development emphasizes the need for robust computational analysis to justify further validation through in vitro and in vivo experiments.

## 1 INTRODUCTION

Tuberculosis (TB) is a contagious infection caused by the bacterium *Mycobacterium tuberculosis* (MTB). It primarily originates in the lungs but can potentially spread to other parts of the body, including the brain and spine. In 2018, World Health Organization reported that about 1.7 billion individuals of the world's population (23%) are projected to possess a latent TB infection, which indicates a risk of developing active TB during their lifetime.^1^ Here there were 330,000 cases of multidrug resistance (MDR) and rifampicin resistance (RR) among notified TB patients. According to surveys conducted in Azerbaijan, Bangladesh, Belarus, Pakistan, South Africa and Ukraine, the average level of resistance to all first‐line anti‐TB drugs, including rifampicin, isoniazid (INH), pyrazinamide (PZA), and ethambutol, in new and previously treated TB cases was 19% (95% confidence interval [CI]: 18%–20%) and 43% (95% CI: 40%–46%), respectively. Approximately 1.7 billion people are thought to have latent TB infection, leading to an increased risk of developing TB disease in their lifetime. Pakistan, India, the Philippines, Indonesia and China account for 56% of the global TB burden.^1^ MTB is widely distributed in a latent state in the alveolar macrophages of infected patients. Approximately 5%–10% of these bacteria overgrow from their latent to active state.^2^, ^3^ In addition, co‐infections such as TB‐HIV, immunosuppressants, and age may increase the risk of developing an active TB.^4^ PZA currently stands as the sole drug capable of eliminating *M*. *tuberculosis* in its latent state, effectively reducing the duration of TB treatment from 9 to 6 months.^5^ Combining PZA with rifampin (RIF) and INH constitutes a significantly more efficient and rapid therapy against chronic bacteria.^6^ The pncA gene of *M*. *tuberculosis* encodes pyrazinamidase (PZase), responsible for converting the prodrug PZA to pyrazinoic acid (POA). In environments with very low pH, POA exhibits the capability to suppress the growth of latent Mtb.^7^

Regrettably, the emergence of PZA‐resistant strains of *M*. *tuberculosis* poses a significant obstacle in the treatment of TB, necessitating the identification of innovative therapeutic drugs. The development of resistance to PZA is attributed to genetic mutations in three specific genes, namely pncA, panD, and rpsA.^8^, ^9^ Among these genes, mutations in both the coding and promoter regions of the pncA gene are primarily responsible for 72%–99% of PZA resistance.^10^, ^11^ It is worth noting that the mutation at position G97D is considered a high‐resistance mutation, exerting a substantial influence on the enzyme's activity. This mutation modifies the structural stability, solubility, functional stability, and protein expression level.^12^, ^13^ From a structural perspective, an amino acid substitution can have drastic effects on the protein structure and function, particularly in the active site or distal sites near binding pockets.^13^, ^14^, ^15^ The induced mutation may also exert a distal impact at a long‐range position from the active site.^16^ Exploring such distal site impacts of the mutant on the active site or nearby regions provides valuable information for a better understanding of the phenomenon. However, these analyses are time‐consuming and expensive to address solely through experimental procedures. As an alternative to experimental methods, molecular modeling simulations in the context of classical and quantum chemical simulations have been extensively used to explore mechanisms, including those related to protein–drug interactions and protein‐associated mutations. This approach is particularly valuable in understanding drug resistance mechanisms caused by mutations and the ensuing consequences for various diseases.^14^ In comparison with experimental methods, molecular dynamics simulation (MDS) have an advantage in exploring the detailed mechanisms of drug resistance as well as in analysing protein interaction networks, predicting drug targets, predicting binding sites and binding affinity at low cost and with low resource input (10.55522/jmpas.V11I3.2300).

The utilization of computational methodologies is pivotal for the discovery and optimization of novel pharmaceutical agents, offering a promising avenue for substituting existing medications, thus mitigating development costs and risks. Tools like FAF‐Drugs4, ADMETlab2, Pred‐hERG, pkCSM, vNN‐ADMET and PreADMET are adept at forecasting pharmacokinetic and toxicological attributes, prioritizing the safety profile of drug candidates (10.3390/molecules28020776). In silico techniques facilitate the exploration of chemical reactions and the performance of virtual screening, enriching our understanding of structural, chemical, and binding interactions within theoretical ligand‐receptor complexes as well as biological aspects that are critical for the development of targeted therapeutics (10.3389/fchem.2018.00030). Computational analyses are also invaluable for uncovering new therapeutic potentials by amalgamating diverse data sets, including transcriptomics and electronic health records, which expedites the drug discovery and development timeline while diminishing dependence on animal testing (10.1002/jac5.1519). Furthermore, advancements in Artificial Intelligence, particularly machine learning, have significantly propelled drug design forward by enabling the prediction of biological effects and supporting the creative generation of new drugs for specific molecular targets (10.1038/s41573‐023‐00774‐7).

In this study, we explored the mechanism of PZA resistance associated with the G97D mutation in the Mtb PZase enzyme using a comprehensive computational framework. Novel compounds with high binding affinity to mutant forms of Mtb‐PZase were identified and characterized using a range of molecular modelling techniques, including pharmacophore‐based virtual screening, docking and molecular dynamics (MD) simulations. In addition, pharmacoinformatic predictions incorporating physicochemical, medicinal chemistry and ADMET considerations as well as quantum calculations of internal energies and reactivity descriptors were used in this study. The results of this investigation provide a hopeful basis for the development of innovative drugs that can combat resistance to PZA in MTB. Consequently, this opens up new possibilities for the improvement of therapeutic approaches which can be further validated by laboratory and animal‐based research.

## 2 MATERIALS AND METHODS

**FIGURE 1:**
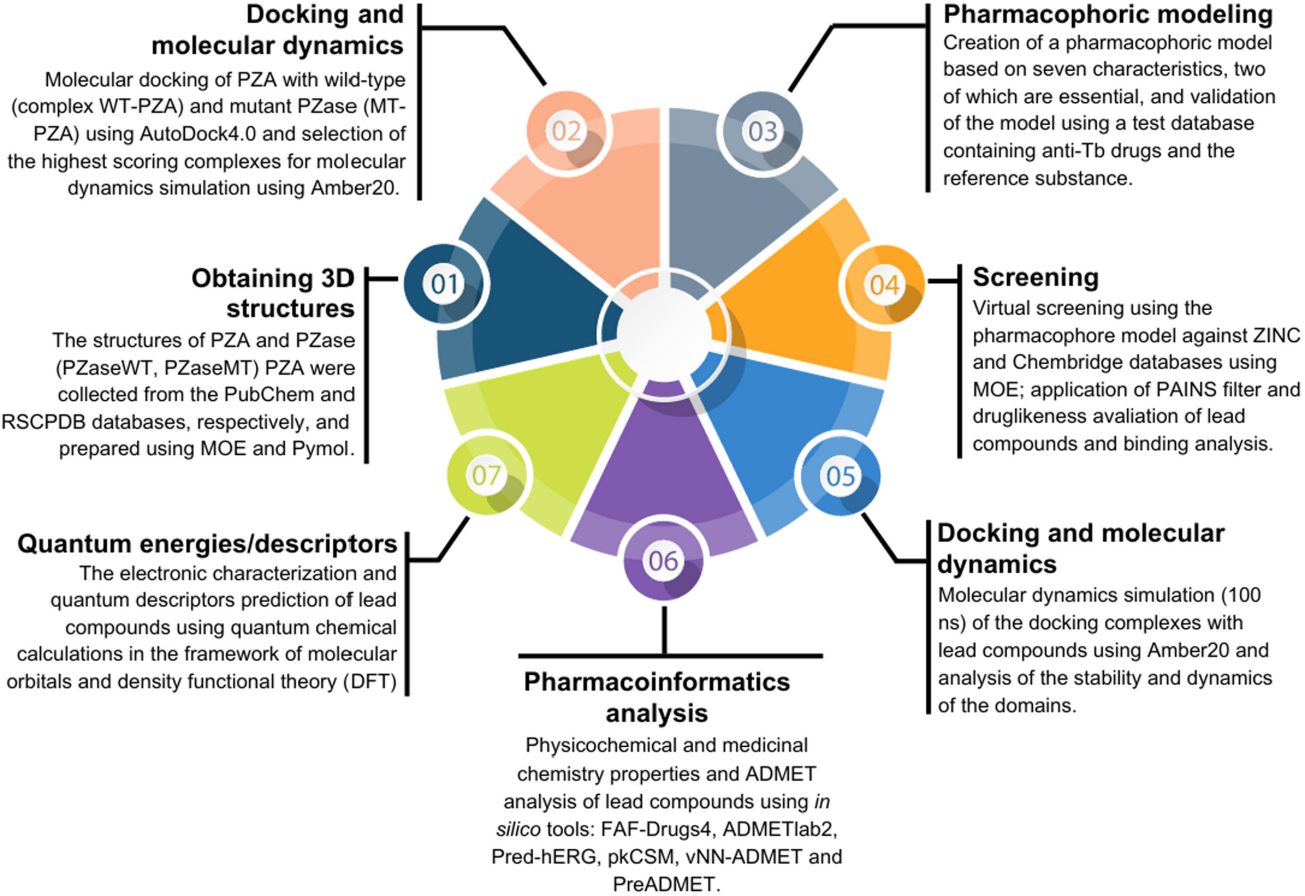
Schematic representation of the overall protocols for this study.

The design of the overall mechanism and the various tools used in this study are illustrated in Figure 1.

### 2.1 Protein (PncA^WT^ , PncA^Mt^ )—Ligand (PZA) docking

The 2D and 3D structures of PZA (PubChem ID: CID1046) and Mtb‐PZase (PDB ID: 3PL1) was taken from the PubChem (https://pubchem.ncbi.nlm.nih.gov/) and RSCPDB (https://www.rcsb.org/) database, respectively. Before starting the research, the water molecules are first removed from the target protein. Then the structure was checked for missing residues or chains. The molecular operating environment (MOE) was used for structure preparation, 3D protonation and assignment of the missing residues. Although certain mutant structures of the enzyme were not available, PyMol was used to create mutations in the enzyme structure at specific positions.^16^ The tool MOE was used to minimize the ligand structure. The PZA was then saved in format (rec.mdb) using file‐new‐database in MOE main windows. The molecule (PZA.mdb) was docked to the binding pocket of the wild type (PZaseWT) and mutant (PZaseMT) enzyme PZase using AutoDock4.0.^17^ First, the wild‐type PZase docked to the PZA drug (WT‐PZA), then the mutant PZase docked to the drug (MT‐PZA). By default, the cluster RMSD was set to 3 Å and the docking type to a protein small ligand. The complexes with the highest score were saved in.pdb format and then subjected to MD simulation using Amber20. Visualization of the interactions was verified in a molecular working environment.

### 2.2 Molecular dynamics simulation

The most widely used computer simulation method, MD, allows us to obtain a wealth of information about the stability of protein–protein, protein–ligand, and protein–nucleic acid complexes.^18^, ^19^ MDS were performed to study the stability of the drug PZA and domain dynamics. The Amber20 software package was used to perform the MD analysis.^20^, ^21^, ^22^ The FF14SB force field was used to define the force field parameters for the protein. A total of four systems wild type (WT), WT free, mutant type (MT) and MT frees were applied to the MDS. The total number of charges of the designated complex was identified and then the charge of the systems was neutralized. The complex was neutralized with counterions after being placed in a cubic box filled with TIP3P‐specific water representation. To avoid unfavourable interactions, steepest descent minimization was used followed by two‐step equilibration to construct the initial structures for the production simulations. Position constraints were applied to all atoms during equilibration to check any configuration changes. Finally, production MDS were performed for 100 ns without any constraints. The CPPTRAJ module was used to identify all trajectories using Amber20.

### 2.3 Pharmacophore modelling

**FIGURE 2:**
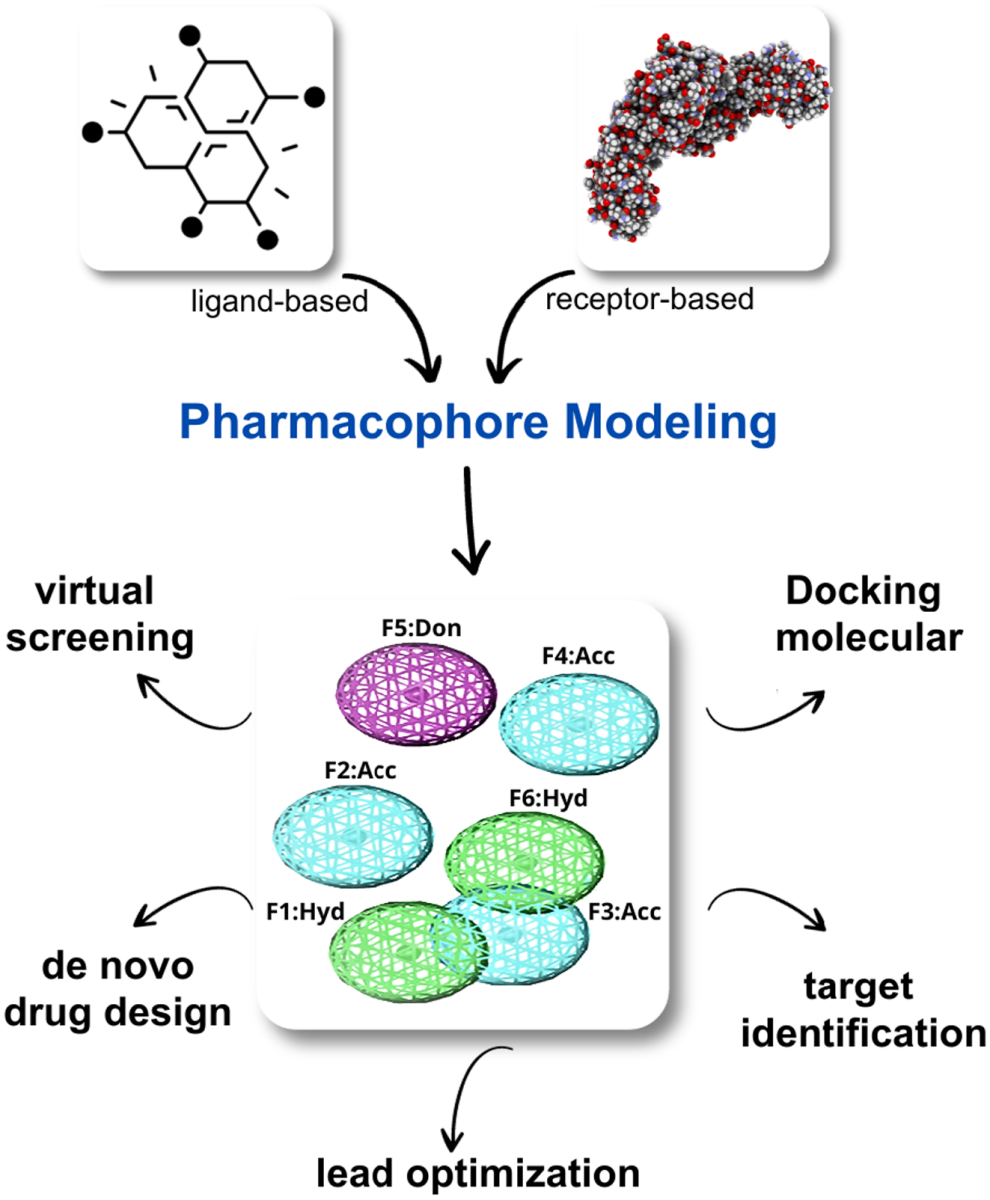
Protocol for pharmacophore and its application in drug discovery.

A pharmacophore is a 3D representation of the steric (shape) and electronic (charge) features of a molecule that are essential for its interaction with a particular target protein or receptor. Using a pharmacophore model, researchers can identify compounds that share similar steric and electronic features that are critical for blocking or modulating the activities of the target protein. The purpose of the pharmacophore in this study is to identify new compounds that have the best pharmacophore properties that are critical for biological properties toward the target proteins.^23^ The pharmacophore was designed with seven features, containing three hydrogen bond acceptor (HBA), two hydrophobic, one aromatic, and one H‐bond donor (HBD) features were prioritized for generation of pharmacophore model two of which were considered essential. A test database of anti‐TB drugs and the reference compound was used for model validation. The validated pharmacophore model was applied to search databases or compound libraries (TCM and Cambridge) for potential hits or new molecules that matched the pharmacophores features. The detailed protocol for the complex‐based pharmacophore is shown in Figure 2.

### 2.4 Chemical databases are used to evaluate drug‐like features

The verified pharmacophore was then matched to the ZINC and Cambridge databases using a virtual screening protocol using in MOE version 2020 to analyse and monitor drug‐like properties. In silico screening, such a method is constructed as a 3D query to select the ideal hits for specific physicochemical parameters. The main goal of the evaluation is to find a new drug‐like pose that can be evaluated later.^24^ After virtual screening, Lipinski Rule of Five (Ro5) was applied to evaluate the drug similarity of the compounds. Lipinski Ro5 is a set of guidelines used in drug discovery to evaluate the likelihood of a compound's success as a drug based on its physicochemical properties. According to this, a drug‐like compound would have a log P (0.4 to +5.6), a molecular weight (180–500) kDa, and multiple atoms in the range of 20–70 (less than 5 hbond don and no more than 10 hbond acc), and a polar surface area no greater than 140.^25^

### 2.5 Molecular interaction analysis and lead compound selection

Molecular interaction analysis and lead compound selection are critical steps in the drug discovery process. These steps involve screening potential drug candidates for their interactions with the target protein or receptor of interest to identify promising lead compounds that can be developed into potential drugs. Molecular docking was performed with standard parameters that is the model protonation state was established based on the neutral pH, partial charges were added, and the MMFF94x force field was used to minimize the model 3D coordinates until the RMSD gradient was 0.1 kcal/mol/Å^2^. The GBVI/WSA rescoring method (to get the refine confirmation from the docked poses), the London dG scoring function (to calculate the binding free energy of the molecule), and the Triangle Matcher Docking algorithm (The Triangle Matcher method was used as the placement method which generates poses by the triplet of ligands aligning, and the maximum number of poses were generated for small compound) methods were used. A maximum of five poses were allowed to be recorded for each ligand during the docking process. The calculated root mean square deviation (RMSD) between the conformation in the co‐crystal and the conformation redocked using the SVL script from MOE was 0.78 Å. This RMSD value indicates that the docking approach used is reliable and capable of making accurate predictions of ligand binding in the active site of the PncA mutant.^26^ Based on the docking score, binding interactions, and binding affinity of the molecular docking results, a total of five compounds were selected to rank high. Among them, two compounds were selected from the ZINC database and three compounds were selected from the Cambridge database. These compounds showed the most promising properties in terms of their potential to bind effectively and with high affinity to the active site of the PncA mutant.

### 2.6 Analysis of physicochemical, medicinal chemistry and ADMET properties

In this study, a variety of in silico tools were selected to investigate the physicochemical and medicinal chemistry properties of the ligands, focusing on their ADMET profile. The selection of these tools was supported by their demonstrated validity and extensive validation in the scientific literature.^27^, ^28^, ^29^ FAF‐Drugs4, Lipinski violations, Veber rule, Egan rule, solubility forecast index, Het/carbon ratio, and total charge analysis, served as a fundamental tool for preliminary filtering of the compound libraries.^30^, ^31^, ^32^ ADMETlab2 was used to extend the analysis to cover a range of physicochemical and medicinal chemistry properties associated with FAF‐Drugs4. It was important to investigate blood–brain barrier permeability (Log BBB), fraction unbound (Fu), plasma protein binding (PPB), and several other important properties such as volume distribution (VD) in humans (log L/kg) and interactions between cytochrome P450 substrates and inhibitors, which are central to a sophisticated understanding of the behaviour of ligands in biological systems.^33^ The Pred‐hERG app, used specifically for cardiotoxicity analysis, played a critical role given the significant importance of cardiac safety in drug development.^34^ The pkCSM tool, which was used in the evaluation of a variety of properties such as water solubility (log mol/L), Caco_2_ permeability (Log Papp in 10), absorption in human intestine (HIA) (% absorbed), provided indispensable insights into the solubility, permeability and potential hepatotoxicity of the ligands, which are critical for the evaluation of their drug and toxicity profiles.^35^, ^36^, ^37^ The vNN‐ADMET tool was used for a comprehensive toxicity assessment that included human liver microsomal (HLM) stability, cardiotoxicity, cytotoxicity, MMP (mitochondrial toxicity), and Ames toxicity/mutagenesis, and contributed significantly to characterize the safety profile of the ligands.^38^, ^39^ Finally, the PreADMET tool was used for its capabilities in MDCK permeability analysis and carcinogenicity assessment along with Salmonella strain mutagenicity assessment, which was essential for assessing the potential genotoxic and carcinogenic risks of the ligands.^40^, ^41^

Using these tools together allowed for a robust and multidimensional analysis of the ligands, with each tool complementing the others, making the study even more comprehensive and rigorous. The nuanced insights that emerged from these analyses greatly enhanced the understanding of the ADMET profiles of the ligands and other key properties and underpinned the methodological rigour required for a scientific endeavour such as this.

### 2.7 Pan assay interference compounds

Pan assay interference compounds (PAINS) is an electronic filter that focuses on the quality of compounds in the database. PAINS screens compounds that are more likely to interfere with assays and are extremely reactive, as well as molecules that are frequently hit and are unfamiliar with toxicophore filters. Therefore, it is suggested that a combination of filtered compounds be examined to obtain the desired pharmacokinetic properties such as ADMET of a chemical sooner in the drug design and development phase.^42^ PAINS‐remover (https://www.cbligand.org/PAINS) is designed and constructed to remove the PAINS from screening libraries and for their exclusion in bioassays.

### 2.8 Quantum energies and quantum chemical descriptors using molecular orbital calculations

The electronic characterization of five lead compounds using quantum chemical calculations in the framework of molecular orbitals (MO) and density functional theory (DFT). The 6–311 + G (d, p) basis set for quantum chemical calculations, which ensures triple zeta quality in valence space and polarization functions with double zeta quality. This basis set is essential for the accurate representation of MO as linear combinations of atom‐centered basis functions. The need for polarization functions is particularly pronounced for the correct representation of hydrogen bonds, a feature covered by our chosen basis set. It also contains diffuse functions for accurate anion modelling that captures electron density in regions distant from the nucleus. This choice ensures the accuracy and reliability of our calculations, as the 6–311 + G (d, p) basis set has been proven to predict the electronic structure, binding interactions with biological receptors, and spectroscopic and thermodynamic properties of a variety of organic molecules.^19^, ^43^, ^44^, ^45^, ^46^ In addition, a dielectric constant was included through the conductor‐like polarizable continuum model (CPCM) to mimic the influence of the electrostatic environment of the drug and improve the calculations.^19^, ^47^

The following values were calculated to electronically characterize the compounds: total energy (the total energy of the molecule), binding energy (the interaction energy between all the atoms in the molecule, also known as cohesive energy), highest occupied molecular orbital (HOMO) energy (the energy of the highest occupied molecular orbital), lowest unoccupied molecular orbital (LUMO) (the energy of the lowest unoccupied molecular orbital), band gap (the energy difference between LUMO and HOMO), dipole mag (the magnitude of the dipole moment), dielectric energy (the energy stored in the dielectric in a solvated system. It is always equal to the solvation energy of the molecule), solvation energy (the solvation energy calculated as the difference of the total energy of the molecule in solvation and the total energy of the molecule in vacuum), surface area (the solvent accessible surface area of the molecule), cavity volume (the volume of cavities in the solvation calculation), ESP/Muliken/Hirshfeld charges the atomic charges calculated from the electrostatic potential/Muliken method/Hirshfeld method. Key quantum mechanical parameters and descriptors, including the energies of the HOMO and LUMO, the GAP energy (HOMO–LUMO), ionization potential (*I*), electron affinity (*A*), chemical hardness (*ɳ*), softness (*σ*), chemical potential (*μ*), electronegativity index (*χ*) and electrophilicity index (*ω*) were meticulously examined. Such parameters are pivotal for comprehending the extent of ligand interactions within the receptor binding cavities.^48^ The mathematical formulations for these parameters are delineated as follows^49^:

(1) $$\mathrm{GAP}=\varepsilon_{\text{HOMO}}-\varepsilon_{\text{LUMO}}$$

(2) $$I\approx -\varepsilon_{\text{HOMO}}$$

(3) $$A\approx -\varepsilon_{\text{LUMO}}$$

(4) σ=1/η

(5) $$\eta \approx ½\;\left(\varepsilon_{\text{LUMO}}-\varepsilon_{\text{HOMO}}\right)\approx ½\;\left(I-A\right)$$

(6) $$\mu \approx -½\;\left(\varepsilon_{\text{HOMO}}+\varepsilon_{\text{LUMO}}\right)\approx -½\;\left(I+A\right)$$

(7) χ≈−μ≈(I+A/2

(8) $$\omega \approx \chi^{2}/2\eta$$

## 3 RESULTS

### 3.1 Mechanism of binding of Fe^2+^ and pyrazinamide to the wild type and mutant type receptor

**FIGURE 3:**
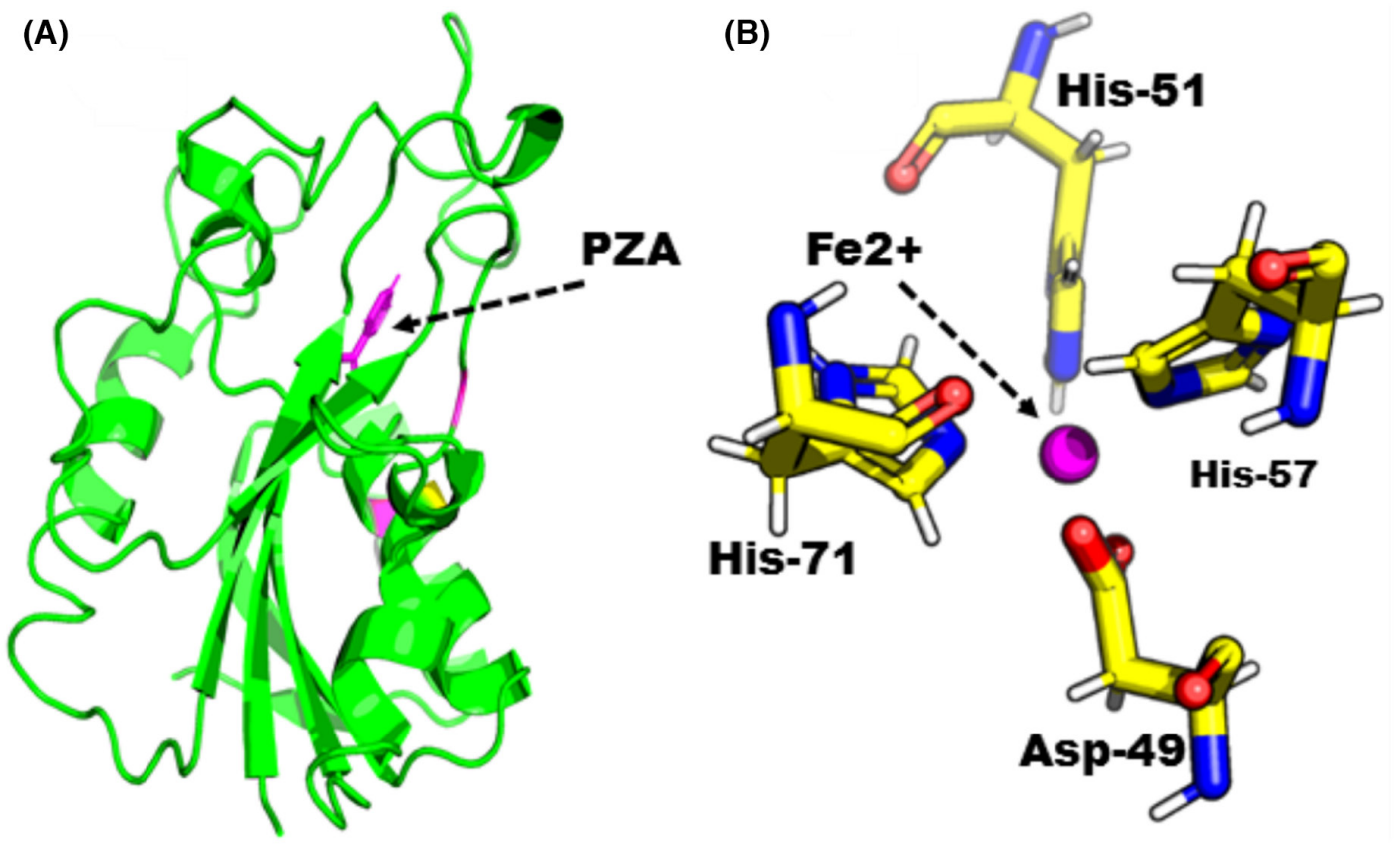
(A) Structure of Mtb PZase and PZA drug. (B) Binding mechanism of Fe^2+^.

The Apo structure of PZase (ID: 3PL1) was downloaded from the PDB and submit to a mutagenesis module using PyMol software, targeting a mutation site to check the indirect effects of the mutation (G97D) on the entire Mtb‐PZase protein. The structure of WT and MT was first energetically minimized using MOE before molecular docking was performed to remove poor contacts between mutant residues and between other residues. A molecular docking approach was used to dock the PZA drug to the active site of Mtb‐PZase. The complex structure of the PZA drug in the active site of Mtb‐PZase (MT‐PZA) is shown in Figure 3A, and the binding mechanism of Fe^2+^, especially the strong interaction with Asp‐49, His‐51, His‐57 and His‐71, is shown in Figure 3B.

**FIGURE 4:**
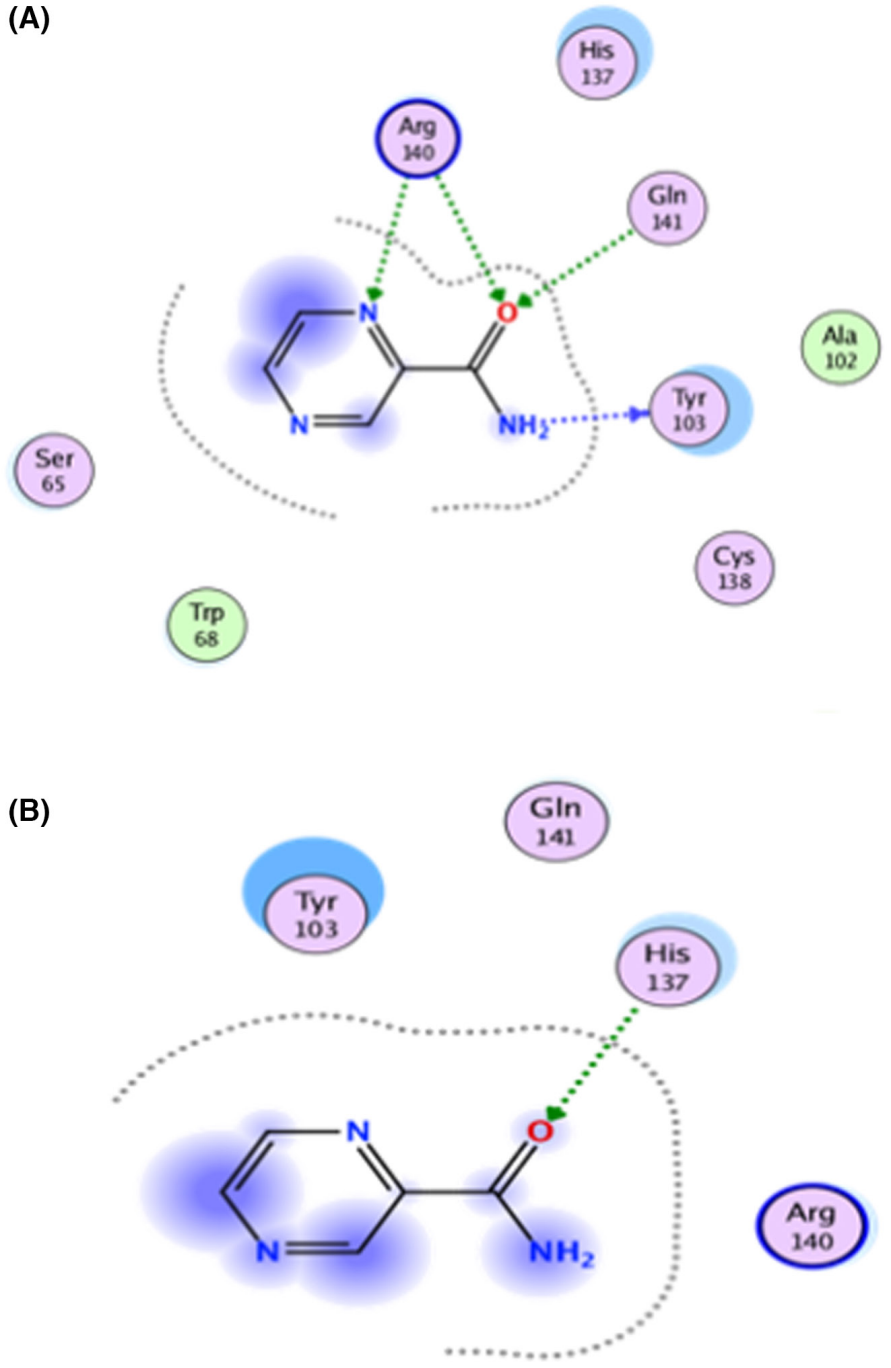
(A) 2D interaction of wild type showing major interactions with Arg140, Gln141 and Tyr103. (B) 2D interaction of MT (G97D) showing interaction with His137.

**FIGURE 5:**
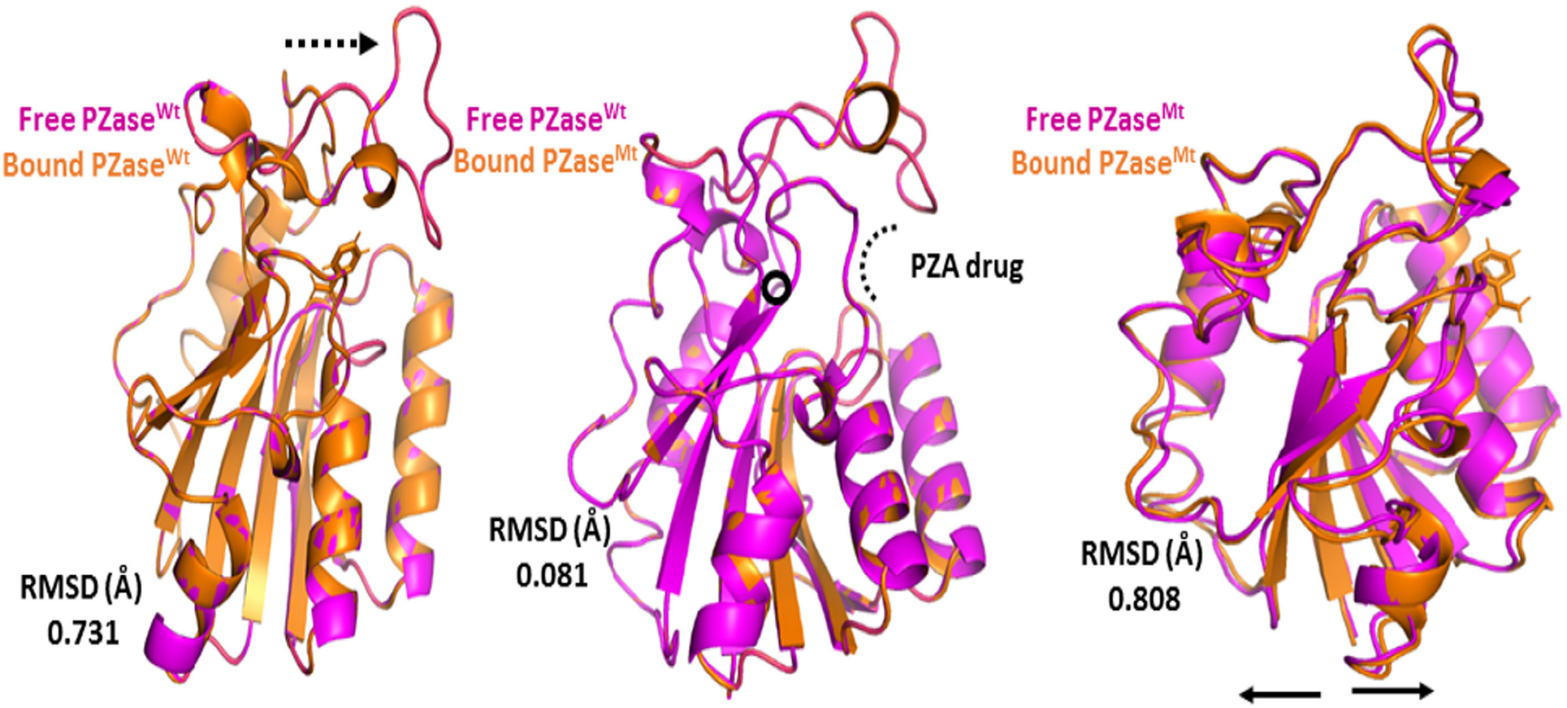
The aligned protein structure of Mtb PZase in free and bound form with the drug PZA the post MD protein RMSd were calculated in each structure. Magentas and orange colour represent different structures. The mutation (G97D) is printed in a small black circle. The movement of the two loops is shown by arrows.

In comparison, PZA significantly reduced the interaction with active site residues in the bound Mtb PZaseMt (G97D) system, as shown in the 2D interaction in Figure 4. The docking score of PZA with both WT and MT PZase is in the following order: wild type (−9.8 kcal/mol), G97D (−6.6 kcal/mol). This shows that the PZA drug has a higher binding affinity for the wild‐type PZase enzyme compared to the mutants (Table 1).In the free Mtb‐PZaseMT and bound Mtb‐PZaseMT systems, the interaction of the PZA drug decreases significantly and the mutation site (G97D) was found at a distance of about 20 (Å) from the binding site (Arg140, Gln141 and Tyr103) of the PZA drug (Figure 5). Upon induction of the mutation in the protein structure, it was discovered that the altered residues had an indirect effect on neighbouring loops (α‐helix1 and α‐helix2) adjacent to the PZA binding site.

**TABLE 1:** Docking scores of wild and mutant (G97D).

S. no.	Complex	Docking score	Outcome
1.	Wild	−9.8	Highly stabilizing
2.	G97D	−6.6	Highly destabilizing

### 3.2 Molecular dynamic simulation

**FIGURE 6:**
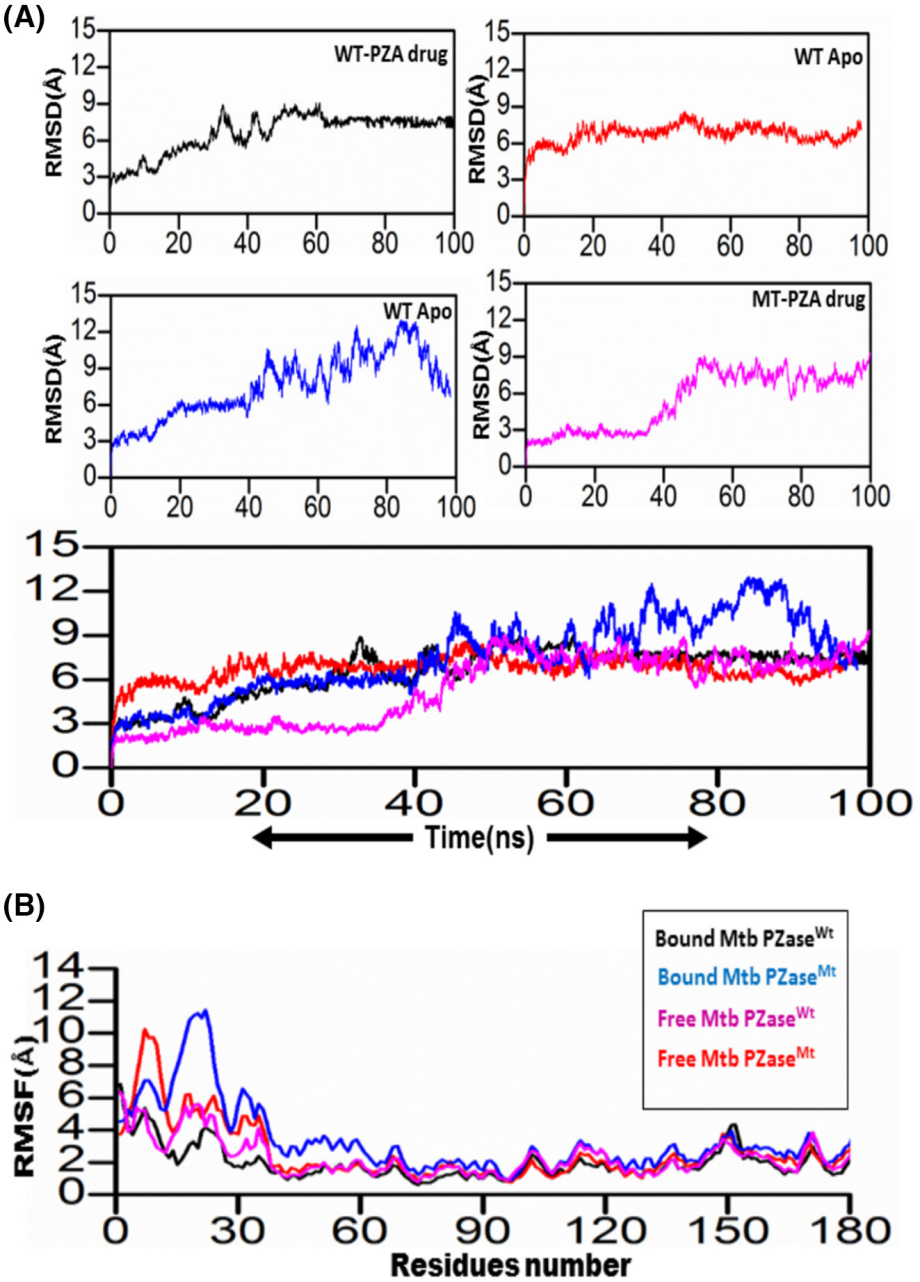
(A) Study of RMSD for all the four systems, black (WT‐PZA drug), red (WT Apo), blue (Mt Apo), and pink (MT‐PZA drug). The comparison of stability can be estimated from the last graph. (B) RMSf for all the four systems. The *X*‐axis indicates the residual number, and the *Y*‐axis shows the RMSf value in Å.

MDS of all four systems were run for 100 ns to verify stability. To check the stability of the structure, several structural properties, RMSd, were calculated for all four systems. First, the RMSd value of the WT‐PZA complex (bound Mtb PZaseWT) increases up to 8 Å, which can be observed up to 65 ns. After the 65 ns, the RMSd value remains stable until 100 ns. For the WT systems, a small fluctuation between 30–40 and 45–50 ns was studied, showing that the remaining system is stable until 100 ns. Compared to MT‐PZA (bound Mtb PZaseMT), the RMSd value first increases to 3 Å, which can be observed until the 40 ns simulation. After that, the RMSd value increases to 9 Å until the last 100 ns simulation. The MT‐PZA shows a high fluctuation throughout the simulation. In the case of WT Apo (free Mtb PZaseWT), the structure shows a stable RMSd pattern between 25 and 50 ns. The overall average RMSd value observed for the 100 ns simulation remained at 3.5 Å. In the case of MT‐Apo (free Mtb PZaseMT), the RMSd value remains at 3 Å until 15 ns, but then gradually increases to 12 Å until the last 100 ns simulation. The mechanism of stability of the MT‐PZA complex indicates a lower binding affinity of the PZA drug to the active site and shows the limited interaction (strength of H‐binding) with the PZA drug, demonstrating the phenomenon of resistance to the receptor active site (Figure 6A).

To check the effects of residual flexibility of the wild type and mutant (G97D) including (free WT and MT), we analysed the root mean square fluctuation (RMSf). A flexibility pattern was observed in all four systems, but still a difference in the values can be seen. The overall lower flexibility value was observed for WT. The flexibility value for all systems shows some variation in the fluctuation pattern up to 30 residues and after 30 residues lower RMSf values were observed. As a result of the G97D mutation, the MT‐PZA system shows a slightly higher fluctuation pattern up to some regions, as shown in Figure 6B. The result suggests that the mutant exhibits high fluctuation upon binding the PZA drug, which has a destabilizing effect on the residues and internal dynamics.

### 3.3 Pharmacophore validation and examining drug‐like characteristics

**FIGURE 7:**
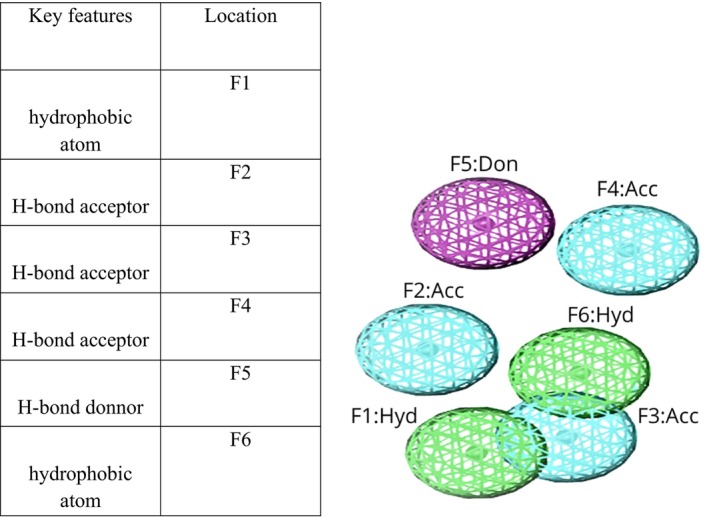
The pharmacophore model.

The test database created a pharmacophore model and a validated 3D model. Interacted closely with the mutant state of PncA, potentially causing the protein in question to stop functioning. We created a pharmacophore model that includes a total of seven key features. These features consist of two hydrophobic atoms (F1 and F6), three H‐bond acceptors (F2–F4), and one H‐bond donor (F5). Figure 7 illustrates the pharmacophore model we developed.

### 3.4 Chemical libraries for drug‐like features evaluation

A validated pharmacophore model was used to screen the ZINC and Chembridge libraries. As a result, 107 structurally dissimilar hits were identified in the ZINC library and 732 hits in the Chembridge library. Subsequently, 07 properties were found to be compatible with the extended Huckel Theory pharmacophore model. Further refinement using Lipinski's Rule of Five (Ro5) resulted in the selection of 85 hits from the ZINC library and 420 hits from the Chembridge library. Ultimately, this approach yielded several chemical compounds that exhibited stronger interactions with the target compared to the PZA compound.

### 3.5 The interaction model of a protein–ligand complex

**FIGURE 8:**
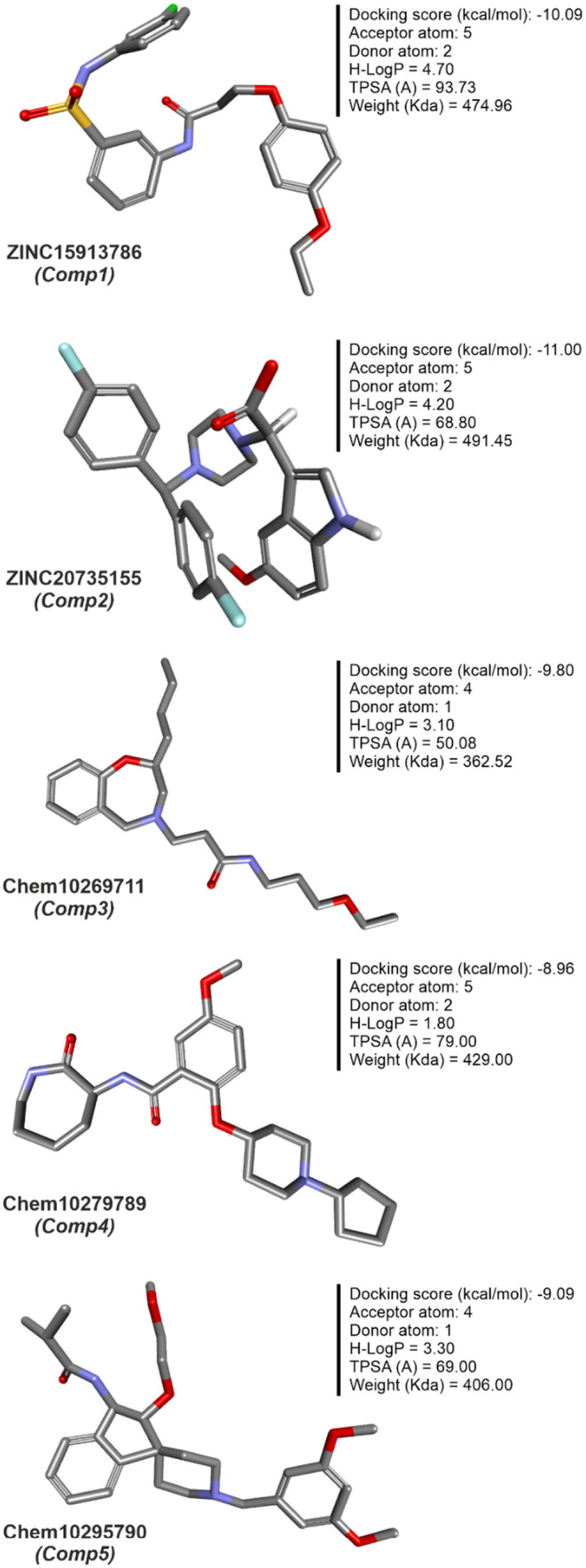
Schematic representation, molecular and docking properties of lead compounds.

**FIGURE 9:**
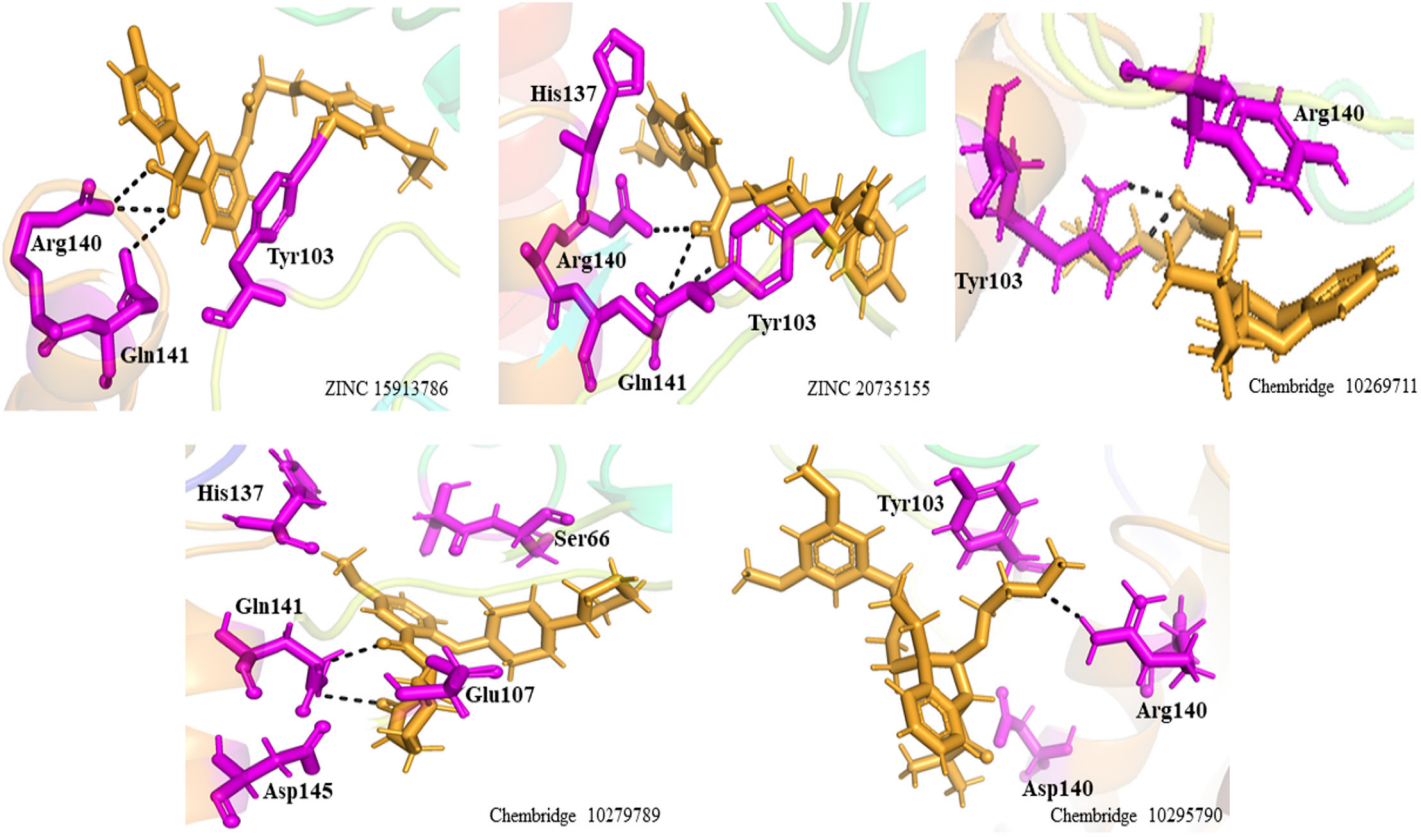
Binding interactions of the lead compounds with the protein target.

The interaction model of a protein‐ligand complex can be constructed using the docking approach. We docked 85 and 420 molecules from the ZINC and Chembridge libraries, respectively, to the mutant form of PncA (G97D). Each library provided many hits from which the five highest scoring conformations of all docked compounds were selected, including results of PAINS Filter assay (Figures 8 and 9). In the ZINC database, ZINC15913786 (ZINC20735155) has a docking score of −10.09 (−11.00), five^5^ acceptor atoms, two^2^ donor atoms, 4.70 (4.20) H‐log P, and MW of 474.96 (491.45) kDa. In the Chembridge database, Chem10269711 has a docking score of −9.8, 4 acceptor atoms, one donor atom, a weight of 362.52 kDa, and an H‐log P of 3.1. In addition, Chem10279789 (Chem10295790) has a docking score of −8.96 (−9.09), five^4^ acceptor atoms, two^1^ donor atoms, a weight of 429 (404) kDa, and an H‐log P of 1.8 (3.3). Additionally, results of PAINS filter assay indicate lead compounds have higher docking score, binding mode, pharmacophore mapping, binding energy (stability), binding affinity, visible ligand interaction and better ADMET properties. Among the most perfidious are the eight listed in Table 2, whose reactive portion shown in brackets.

**TABLE 2:** The areas enclosed and red colour parts make these chemistries as artefacts.

PAINS	Reason of being PAINS
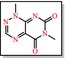	*Toxoflavin* **Redox cycler:** can produce hydrogen peroxide, which can activate or inactivate different proteins
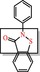	*Isothiazolones* **Covalent modifier:** reacts chemically with proteins in non‐specific, non‐drug‐like ways
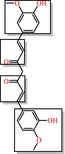	*Curcumin* Covalent modifier, **membrane disruptor:** muddles response of membrane receptors
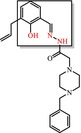	*Hydroxyphenyl hydrazones* Covalent modifier, **metal complexer:** sequesters metal ions that inactivate proteins
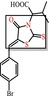	*Ene‐rhodanine* Covalent modifier, metal complexer.
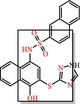	*Phenol‐sulphonamides* Redox cycler, covalent modifier and **unstable compound** breaks down into molecules that give false signals
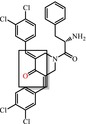	*Enones* Covalent modifier
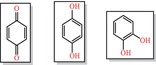	*Quinones* AND *Catechols* Redox cycler, metal complexer, covalent modifier

### 3.6 Physicochemical and medicinal chemistry analysis

**FIGURE 10:**
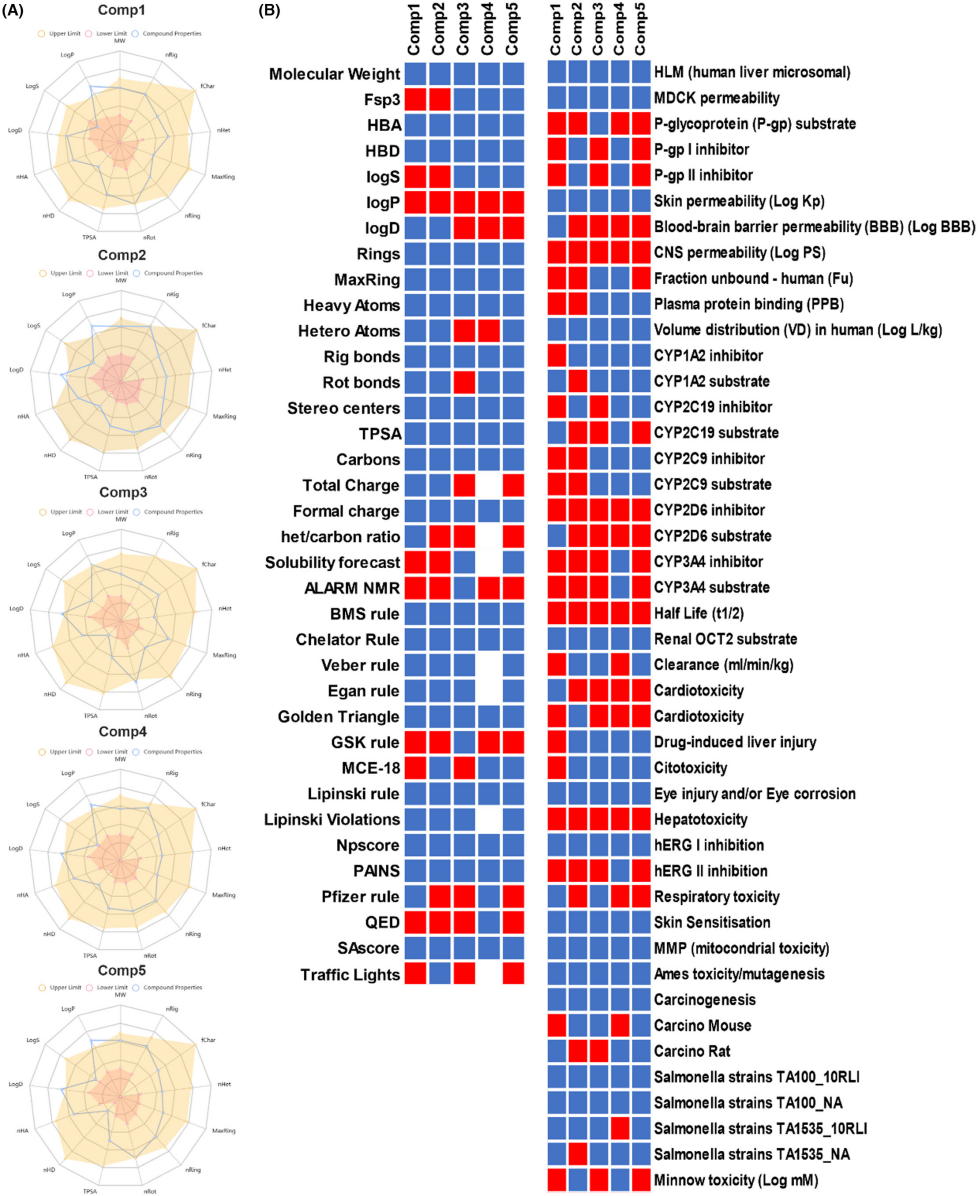
(A) Physicochemical, medicinal chemistry and (B) ADMET analysis of lead compounds.

Analysis of chemical compounds from the ZINC and Chembridge libraries, as described in the previous section, has revealed several molecules with promising therapeutic potential. However, their physicochemical properties and medicinal chemistry profiles remain critical for their future development as drug candidates. The compounds, mainly Comp1, Comp3, Comp4 and Comp5, exhibit different profiles (Figure 10). Some compounds show more favourable properties in certain parameters, while others are superior in different areas. This discrepancy suggests the potential for a combinatorial or synergistic approach in future studies, possibly using more than one compound for improved therapeutic efficacy.

An assessment using the pharmacophore called attention to potential toxicity issues. All compounds, except Comp2, could have some degree of toxicity. Therefore, experimental toxicological testing is of utmost importance prior to any clinical research.

Solubility remains a critical factor in the oral bioavailability of a drug. In this context, Comp3 and Comp5 emerge as promising candidates based on the solubility prediction index. In contrast, Comp1 and Comp2 may require additional strategies, such as formulation with nanocarriers or the use of prodrugs, to improve their solubility. It is encouraging to note that none of the compounds studied violated Lipinski's rules, and this observation underscores their potential as drug candidates. However, the violation of other rules, such as those of GSK and Pfizer, by a few compounds suggests potential limitations in certain therapeutic applications. The data also suggest the potential for molecular optimization. For example, optimization of Comp1 could improve its three‐dimensionality, and refinement of Comp4 could address its structural complexity. Comp3 consistently stands out in our analyses, showing optimal properties in several categories. This profile underscores its position as a prime candidate for comprehensive investigation and possible development. Nonetheless, its overall positive charge warrants consideration in subsequent formulations and interaction analyses. While the present data serve as an informative analysis of the compounds in question, they should be used as a basis for in‐depth investigation rather than as a final determination of a compound's viability.

### 3.7 ADMET analysis

ADMET analysis addresses the pharmacokinetic and toxicological properties of the five compounds (Figure 10), highlighting their potential to combat PZA resistance in *M*. *tuberculosis*. All compounds have shown stability in the hepatic microsomal stability (HLM) assay, a positive indication of drug metabolism. In particular, Comp3 exhibits high MDCK permeability, suggesting an advantage for oral bioavailability. However, the observation that all drugs except Comp3 are substrates for P‐glycoprotein raises concerns about potential problems with absorption and systemic distribution of the drug. In terms of distribution, Comp2 is a promising candidate for CNS targeting because it can cross the blood–brain barrier (BBB), although this was not the goal of this drug. In addition, the low plasma protein binding profile (PPB) of Comp4 means that a greater proportion of the drug remains unbound and bioavailable, which is critical for therapeutic efficacy. These properties, in conjunction with the distribution characteristics of the drug, are critical for determining the therapeutic window and dosing regimen of the drug. The interaction of these compounds with cytochrome P450 enzymes, the major players in drug metabolism, provides insight into their potential metabolic pathways and potential drug–drug interactions. It is imperative that these interactions be further explored, as they may decide both therapeutic efficacy and potential side effects. Of note, the prolonged half‐life observed for Comp3 could result in prolonged systemic exposure, a characteristic that could influence dosing frequency and potential accumulation.

Toxicity evaluation reveals a broad range of risks and safety profiles for all agents, which is a critical factor in their therapeutic suitability. In particular, the assessment of cardiotoxicity described by both vNN‐ADMET and pred‐hERG reveals a diverse risk landscape. Comp3, Comp4 and Comp5 exhibit cardiotoxic potential in both assessments, warranting a thorough investigation of their cardiac safety profile, especially in long‐term therapeutic scenarios. In addition, hepatotoxicity and drug‐induced liver injury (DILI) data suggest that Comp1 is an agent of concern, such that its hepatic safety profile warrants further investigation. This is particularly important because PZase is metabolized via the liver in the treatment of *M*. *tuberculosis*. The carcinogenicity data also highlight the complexity of toxicity profiles, as different risks have been identified in different rodent models. The identification of potential carcinogenic risks with Comp1 and Comp4 in mouse models compared with carcinogenicity data for Comp2 and Comp3 in rat models underscores the need for a comprehensive assessment of carcinogenicity in different biological systems to accurately identify species‐specific and cross‐species carcinogenic risks. In addition, the environmental toxicity data presented in the toxicity tests in *T. pyriformis* and toxicity in fish (minnows) underscore a critical dimension of ecological safety. Comp2 and Comp4 exhibit lower toxicity in the minnow assay, indicating a relatively benign environmental toxicity profile—an aspect that is increasingly becoming a cornerstone in the holistic evaluation of new therapeutics.

The toxicity assessment included in this analysis illuminates the multifaceted toxicological landscape in which these compounds operate. It points to a nuanced, multidimensional framework for toxicity assessment to decipher the full spectrum of safety profiles and support safety‐oriented optimization of drug development, particularly in the search for effective therapeutics against PZA resistance in *M*. *tuberculosis*.

### 3.8 Quantum energies and quantum chemical descriptors

**FIGURE 11:**
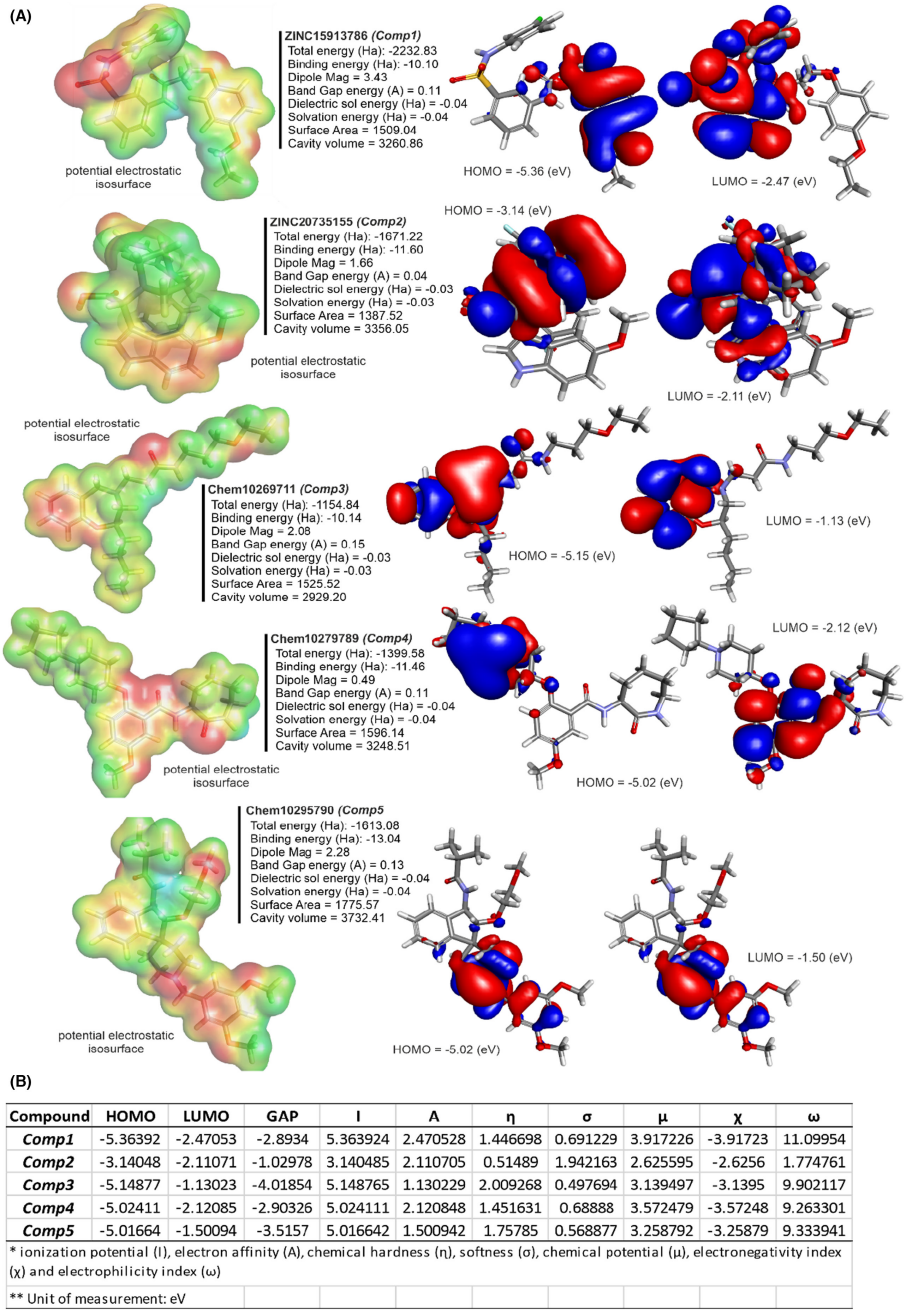
(A) Quantum energies—total energy, binding energy, highest occupied molecular orbital (HOMO) energy, lowest unoccupied molecular orbital (LUMO) energy, dipole mag, band gap energy, dielectric energy, solvation energy, surface area, cavity volume—and 3D visualization of potential electrostatic iso surface, HOMO and LUMO of lead compounds. (B) Quantum chemical descriptors using molecular orbital calculations.

In the electronic characterization of five lead compounds using quantum chemical calculations from MO and DFT, we utilized the 6–311 + G (d, p) basis set, celebrated for its accuracy in determining the electronic structure and binding interactions of organic molecules (Figure 11). The total energy ranged from −2232.83 Ha for Comp1 to −1154.84 Ha for Comp3, with Comp1 displaying the highest energy, hinting at its stability. Binding energies revealed Comp5 to possess the strongest inter‐atomic interactions at −13.04 Ha. Differences were observed in the dipole magnitudes, with Comp1 having the highest at 3.43 and Comp4 the lowest at 0.49. Dielectric solvent and solvation energies exhibited little variation among compounds, with values between −0.04 and − 0.03 Ha. Notably, Comp5 showed the highest solvent‐accessible surface area, indicating its potential for increased interactions, while Comp1 and Comp5 shared the most extensive cavity volume at 3260.86. Diving into the quantum chemical descriptors, the HOMO energy was most prominent in Comp1 at −5.3639, while Comp2's LUMO energy peaked at −2.1107. Comp3 highlighted a significant band gap of −4.0185, indicating a potential difference between its LUMO and HOMO energies, whereas Comp2 registered the smallest band gap. Examining the ionization potential and electron affinity, Comp1 led with the highest values, but Comp3 lagged in electron affinity. Chemical hardness and softness metrics showcased Comp3 as the hardest compound and Comp2 as the softest. Finally, in terms of chemical potential, electronegativity index and electrophilicity index, Comp1 stood out in the former two categories, while Comp5 exhibited the highest electrophilicity. These quantum energies and descriptors are crucial in assessing the compounds' electronic properties, offering invaluable insights into their potential interactions within receptor binding cavities.

## 4 DISCUSSION

TB, a contagious disease that primarily affects the lungs, remains a major global health challenge, exacerbated by the emergence of drug‐resistant strains of *M*. *tuberculosis*. Mutations in the pncA gene of MTB, particularly the G97D mutation, have been identified as the major contributors to this resistance.^1^ The mechanism of this mutation was evaluated using MD simulation including RMSd and RMSf to better understand the effects of stability and residual fluctuation in WT and MT bound systems. A similar study was also performed in the case of W68R, W68G and K96R.^13^, ^50^ Molecular docking study was performed for wild‐type Mtb PZase and showed key interaction with Arg140, Gln141 and Tyr103 in H‐bond formation with PZA. The calculated docking score for wild‐type Mtb PZase was −9.8 kcal/mol and for wild‐type Mtb PZase the docking score was −6.6 kcal/mol with one interaction (His135) (Figure 4). Other mutations associated with this resistance are also found in the literature, such as the S324F, E325K and G341R variants of PZase,^51^ which require a deeper understanding of their biochemical implications. The difference in docking score was further tested for stability of the systems. The RMSd value of the WT‐PZA drug (bound Mtb PZaseWt) increases up to 8 Å, which can be observed until 65 ns. After the 65 ns, the RMSd value remains stable until 100 ns. The RMSd value for MT‐PZA drug (bound Mtb PZaseMt) first increases to 3 Å, which is observed until the 40 ns simulation. Thereafter, the RMSd value increases to 9 Å until the last 100 ns simulation.

We performed pharmacophore‐based virtual screening using commercially available databases, namely ZINC and Chembridge. Subsequently, we obtained 107 structurally distinct hits from the ZINC library and 732 hits from the Chembridge library. Finally, we selected a total of five lead compounds (two from the Zinc database and three from the Chembridge database) based on Lipinski Ro5, docking score, and good interaction. Integration of molecular docking and in vitro studies, particularly in the field of drug discovery for complex diseases, has shown good agreement between the results of the two approaches. This suggests that docking scores can be reliable indicators of drug efficacy in vitro,^52^ that docking scores are a more realistic evaluation target than physicochemical properties, and that they lead to benchmark tasks that are more challenging and more closely related to real‐world problems in drug discovery.^53^

Five PZase single point mutations, namely His82Arg, Thr87Met, Ser66Pro, Ala171Val and Pro62Leu, identified in a MD study by Pitaloka et al.,^54^ were analysed using MD simulations in both the unbound and bound states of PZA. The results indicate that mutation of His82 for Arg, Thr87 for Met, and Ser66 for Pro in PZase affects the coordination state of the Fe^+2^ ion, which is a necessary cofactor for enzyme activity, According to our findings, the mutations found in Dian's study alter the flexibility, stability and fluctuation of amino acid residues Asp49, His51 and His57 around the Fe^+2^ ion, resulting in an unstable complex upon dissociation of PZA from the PZAse binding site. Gatwiri et al.^55^ performed a virtual screening of compounds like anti‐tuberculosis drugs in a ZINC database using the Swiss Similarity tool. The pharmacokinetic and toxicological profiles of the selected compounds were then evaluated using Swiss ADME and Pro Tox Server, respectively. This identified 14 potential compounds for the treatment of multidrug‐resistant tuberculosis (MDR TB) in chronic patients. These tools helped identify and simulate different treatment patterns and strategic interventions and provided information on the potential outcomes and efficacy of different approaches. By integrating data from multiple sources as well as our study, the tools optimize diagnostic and treatment protocols, improving the accuracy and efficiency of TB management.

In addition, Gabriel et al.^28^ highlight the paramount importance of a prior in silico study in the drug approval process, noting the recent global approval of tecovirimate for the treatment of monkeypox (MPX). Reference is made to the paper by Zovi et al. entitled “Pharmacological Agents with Antiviral Activity against Monkeypox Infection,” which acknowledges the efficacy and safety of tecovirimat, although existing safety data are limited. The authors emphasize the need for a comprehensive scientific literature base for human use and present theoretical assessments of the safety of tecovirimat. They point to potential risks, including liver, respiratory and kidney damage, as well as carcinogenic potential. The authors argue for rigorous, well‐structured studies to ensure not only the pharmacologic efficacy of tecovirimate but also its safety in humans. Finally, they express concerns and suggestions for developing a safer treatment protocol for MPXV‐infected patients and emphasize the indispensable role of prior in silico studies in this critical evaluation process. In the context of drug discovery, our research has involved extensive virtual screening in the ZINC and Chembridge databases, which has led to the identification of key compounds with strong binding affinities and favourable ADMET profiles.

These properties are critical for predicting the in vivo behaviour of a drug, and our selected compounds, particularly from the ZINC database, showed promising potential in this regard. The pharmacokinetic profile of these compounds, including stability in liver microsomal assays and permeability in MDCK, demonstrates their potential as viable therapeutic agents. The interaction of these compounds with cytochrome P450 enzymes provides further insight into their metabolic pathways and the potential for drug–drug interactions, which is an important consideration given the multiple drugs frequently used in the treatment of TB. Despite these promising results, our toxicity assessments have highlighted potential risks, particularly with respect to cardiotoxicity and hepatotoxicity. These risks underscore the need for a thorough safety evaluation, particularly for compounds such as Comp1, given the metabolic function of PZase in the liver during treatment of *M*. *tuberculosis*. On a positive note, our environmental toxicity assessments indicate that some compounds, notably Comp2 and Comp4, have relatively low toxicity, consistent with the increasing importance of assessing the environmental impact of new therapeutics.

Among the compounds studied, Comp4 emerges as the most promising candidate for therapeutic applications, mainly due to its balanced quantum chemical descriptors.

This balance indicates a harmonious interplay of stability, reactivity, polarizability, electronegativity and electrophilicity, which often translates into lower toxicity and higher selectivity toward target cells.^56^ The use of quantum descriptors in this study is validated by works such as that of Sifonte et al. (2021), who used quantum mechanics to elucidate the toxicity of metal oxide nanoparticles in human keratin cells.^57^ In addition, Grillo et al. (2023) showed that quantum descriptors can provide valuable insights into the electronic structure of biological macromolecules relevant to processes such as catalysis and protein binding.^58^ However, the intermediate values of Comp4, particularly its second highest chemical potential (*μ*), may indicate possible interactions with DNA or generation of cardiotoxic reactive oxygen species.^59^ In contrast, Comp1, with its increased reactivity and electrophilicity, raises concerns about off‐target effects and potential damage to DNA and cell membranes.^60^, ^61^ Comp2 might have limited cellular penetration and lower binding affinity to target biomolecules due to its pronounced electronegativity. Comp3, which is characterized by pronounced hardness and polarizability, might react with external agents, which could affect its specificity for target cells.^62^, ^63^ Comp5 might be less effective against tumours and more susceptible to metabolic elimination due to its stability and lower reactivity.^64^ Notably, pharmacokinetic analysis revealed Comp3 to be the most suitable and least toxic, underscoring the importance of integrating quantum descriptors with biological data for holistic evaluation.^65^ Liu et al. validated the use of quantum descriptors in predicting and understanding the reactivity of covalent warheads associated with afatinib and demonstrated their utility in developing covalent inhibitors.^66^

The integration of Artificial Intelligence in Drug Design (AIDD) is increasingly recognized as a transformative approach, enhancing the outcomes of our research endeavours ( 10.1021/acscentsci.0c00229, 10.1016/j.drudis.2020.10.010). The computational methods utilized by our research group, through the lens of AIDD, significantly enhance the precision of our predictions. However, it is crucial to emphasize that the strength of AIDD's predictive capabilities must be complemented by empirical validation to ensure the reliability and accuracy of our findings. Moving forward, our study plans to partner with other entities to assess the efficacy of our primary compound, underscoring the importance of collaborative efforts in advancing the field of drug discovery.

## 5 CONCLUSION

Our comprehensive study illuminates the complicated dynamics underlying the resistance mechanism attributed to the G97D mutation in *M*. *tuberculosis*. The mutation is proving to be particularly destabilizing, exerting a profound influence on protein dynamics and, in turn, drug efficacy. Through molecular docking, pharmacophore modelling, and virtual screening with renowned databases such as ZINC and Chembridge, we have identified five promising compounds. These compounds not only exhibit excellent interaction profiles but also show commendable ADMET properties and quantum descriptors, escaping the pitfalls of pan‐assay interference (PAINS). Their therapeutic suitability is further strengthened by adherence to safety and environmental protocols, as demonstrated by our thorough analyses. These findings, in conjunction with the promising lead compounds identified, pave the way for the design and development of effective therapeutics.

Despite the promising findings, this study has several limitations, this research relies only on various computational tools. Additionally, molecular docking simulations may be sensitive to various parameters and choice of software, warranting caution in interpreting docking scores as definitive indicators of compound activity. Furthermore, the study would greatly benefit from conducting in‐depth in vitro and in vivo clinical testing to validate the inhibitory activity and potential therapeutic efficacy of the predicted compounds against *M*. *tuberculosis*.

## AUTHOR CONTRIBUTIONS

**Muhammad Shahab:** Conceptualization (equal); methodology (equal); writing – original draft (equal). **Gabriel Christian de Farias Morais:** Investigation (equal); software (equal); validation (equal). **Shopnil Akash:** Investigation (equal); software (equal); validation (equal). **Umberto Laino Fulco:** Investigation (equal); software (equal); validation (equal). **Jonas Ivan Nobre Oliveira:** Investigation (equal); software (equal); validation (equal). **Guojun Zheng:** Supervision (equal); writing – review and editing (equal). **Shahina Akter:** Resources (equal); supervision (equal); writing – review and editing (equal).

## FUNDING INFORMATION

This research received no specific grant from any funding agency in the public, commercial, or not‐for‐profit sectors.

## CONFLICT OF INTEREST STATEMENT

The authors declare that they have no conflicts of interest.

## Data Availability

The datasets generated and analysed during the current study are available in the Mtb PZase with PDB (PDB Id: 3PL1) repository, and PZA molecule (PubChem Id: CID1046) [https://pubchem.ncbi.nlm.nih.gov/compound/Pyrazinamide]. Raw data generated and analysed in this study are available in a scientific repository (DOI 10.6084/m9.figshare.25226165) to ensure transparency and support future research efforts by providing access to our baseline data and results.
